# Editorial Correspondence of Atlanta Medical and Surgical Journal

**Published:** 1857-01

**Authors:** W. F. Westmoreland

**Affiliations:** Paris


*Editorial Correspondence.*

PARIS, October 15, 1856.

*Dear Doctor*—You will, perhaps, be surprised at the reception of a letter from me, with the above date, from Paris, as you were apprised that it was my intention to have spent some weeks in London before crossing the Channel. But, upon my arrival in England, I found that circumstances, entirely beyond my control, made it necessary for me to reach Paris at an early day. My stay in London was less than a day—by no means long enough for me to have attempted to gather any of the medical news of that great city.

I arrived in Paris on the 9th inst. and, since my arrival, my time has been passed in the reception of friends, noting the wonderful changes in this beautiful city, since my departure two years ago, visiting Hospitals, Medical Societies, &c. &c. I regret to say that I find but little doing here at present, in the way of medicine, as we are in the midst of the annual vacation (between the summer and winter course of lectures, which commences the first of September, and continues, usually, until the first of November,) the only respite of the Faculty from their arduous labors during the year. During the vacation, the hospitals are attended by the *agreges* of the Faculty, and the *internes* or hospital physicians; and as there are but few students in Paris during the two months' vacation, we have but few facilities in the way of hospital or private instruction. The regular winter course, however, will commence on the first November; and in some of the hospitals, perhaps, a few days earlier, and with them the various private courses upon specialities, when the student may study any branch of the medical science that he may fancy, to the very best advantage.

I have attended some one of the various hospitals every day since my arrival in the city, and in all find that the application of carbonic acid, as a local anæsthetic, as suggested and practiced by Prof. Simpson, of Edinburgh, has considerable favor. Prof. Simpson's first experiments with this agent, as you are apprised, were in painful affections of the uterus, and with marked success; nor did he find the injection of carbonic acid into the bladder of those suffering from neuralgia or inflammation of that organ, attended with less happy results. At present, in Paris, this agent is not restricted to any organs or class of diseases, but is indiscriminately applied, as well to the external surface, if there be an abrasion, as a diseased mucous membrane. As a test of the anæsthetic properties of this gas, the following experiment has frequently been made, both here and elsewhere:—Apply a blister to the finger or any other portion of the body; after vesication, remove the epidermis, and if exposed to the air there will be more or less pain; if immersed in oxygen gas, the pain will be greatly increased: but, after either, if the denuded surface be exposed to a current of carbonic acid, all pain will instantly cease. This experiment would suggest the importance of this gas in the treatment of burns.

The soothing effect of some poultices to irritable ulcers, and other inflammations, is now supposed to be due to the carbonic acid, set free by the fermentation of these farinaceous compounds. And, again, the agreeable sensation from an effervescing draught in some diseases of the stomach, is accounted for by the contact of the carbonic acid, set free with the diseased mucous membrane of that organ.

Although I have frequently witnessed the application of this gas, both in diseases of the uterus and bladder, yet I have not, but in one case, been able to follow and note the effect. This was in a young man, twenty-four years of age, in the wards of M. Broca. For two years he has suffered constantly with a chronic inflammation of the bladder. For the past six months he has been in the hospital, and submitted to the most rational treatment, without the least amelioration of his sufferings; so sensitive has been his bladder during the whole of his illness, that he has been forced to urinate every half hour—the least distension giving him excessive pain. After the first injection of carbonic acid, there was a marked amelioration of all the symptoms; after the second injection, made twenty-four hours after the first, the amendment was still more perceptible; and, after the third, the same interval intervening, he was enabled to retain his urine for four hours, something that had not occurred for two years; has less pain than at any time during the attack; complains only of pain in the course of the urethra, the result, I suppose, of the frequent introduction of the catheter.

To illustrate further the striking effects of this gas, I have translated the following report from the *Gazette Hebdomadaire* of this week:—

'Michel Denise, aged fifty years, entered Hotel Dieu the 26th Sept. 1856. She has not menstruated for two years, since which time she has had an abundant leucorrhœal discharge. Since April last, she has suffered greatly with pain in the left iliac region; the pain has not been constant, but re-appeared at short intervals, and with such force that it prevented her occupying one position any length of time, in fact, was so severe that it prevented the possibility of sleep, and greatly affected her appetite. Since the appearance of the pains, she has grown very thin. Upon an examination of her vagina, M. Follin found rather an extensive carcinomatous ulceration of the neck of the uterus. On the 30th of September, he injected into the vagina carbonic acid; and, from the moment that the gas reached the neck of the uterus, she affirmed that the pain entirely disappeared. After the injection, no treatment whatever was instituted, and still there was no return of pain. The patient was up during the day; her appetite returned, and it was evident that her general health was improving. On the 8th of October, she said she had suffered to some extent during the night; another injection was made, since which time she has been entirely free from pain.'

In the same journal there is recorded another case, under the care of M. Follin, presenting the same lesions, with a like happy result. Whether the above and like cases on record, are exceptional or isolated, and whether this is like many other remedies for which much has been claimed—making their noise for to-day, to die to-morrow, time alone must determine. Of one thing, however, I feel pretty sure, that if it will accomplish all that has been claimed for it, which I cannot yet believe, it will prove as great a boon to the physician as chloroform has to the surgeon.

The apparatus for generating this gas is of the most simple character, and in the reach of every physician; and, as there is not the least danger in its application, all may test its virtues for themselves. The most simple mode of generating and applying it is as follows:—Into a common glass bottle, with a large mouth, if convenient, put six drachms of tartaric acid, and a solution of one ounce of bi-carbonate of soda, to seven ounces of water; close the bottle with a cork well adapted, through which there passes a tube for conducting the gas from the interior. To make an application to the neck of the uterus, the tube, if elastic, may now be introduced into the vagina. If this is not convenient, or if it is to be thrown into the bladder, attach a hog's bladder to the end of the tube, and, after filling it with gas, detach it and secure a catheter to the mouth of the bladder thus distended, and it may be injected either into the vagina, bladder, or rectum.

I noticed, at La Charete, a few days ago, a new method of treating effusions of blood, the result of contusions, &c. known as the treatment by *ponctions capillaires.* It appears that at one of the recent meetings of the Societe de Chirurgie, M. Vollemier presented to that body this new method, affirming that, after quite a number of experiments, he was convinced that it was the best plan of treating such accidents. This new method consists of a number of punctures over the seat of the collection, by means of a needle; the blood is slowly discharged through these punctures, without the least danger of the entry of air into the sack. M. Vollemier has recently used a small exploring trocar instead of the needle to make the punctures, but impresses the importance of compression of the walls of the tumor, during the time the canula rests in the sack, to prevent the entrance of air.

He but seldom attempts the removal of the whole of the fluid at one time, but contents himself, when operating with the needle more particularly, to make two or three punctures every day until all is removed. He goes still further, and contends that such punctures are not alone applicable to effusions of blood, but are alike efficacious in the treatment of some varieties of purulent collections, and more particularly abscesses of the lymphatic glands. Of the latter, I have seen one example, and must say, that the result was more satisfactory than by the usual method—a full incision. It was an abscess of one of the inguinal glands. In the same wards (those of M. Broca), I witnessed a puncture of the knee-joint with a small exploring trocar, for the removal of what was supposed to be a serous effusion, but which proved to be purulent, the result of acute arthritis. Very nearly all the liquid was removed, to the great relief of the patient. Firm pressure was made over the distended capsule during the time that the canula remained, to prevent the introduction of air. It is now four days since the puncture; no re-accumulation, and the patient, to all appearances, rapidly recovering.

I attended, to-day, for the first time since my arrival, the Academie de Medicine, and was much interested in a discussion upon the proper plan to be pursued in the treatment of ovarian dropsy. The discussion grew out of a report of a case operated on by M. Barth; the case, as well as the operation, presenting some peculiarities. The peculiarity of M. Barth's operation, consists in an attempt to keep the cyst constantly in contact with the abdominal walls, by means of two punctures and an elastic tube. The operation was as follows:—With a long curved trocar he made a puncture above the pubis, the bladder being previously emptied; after the discharge of a small quantity of liquid, he introduced the trocar in the canula, and made a second puncture from within outwards, the trocar passing out some three or four inches above the first puncture; withdrawing the trocar a second time, he, by means of a long needle, passed a tube of india-rubber through the canula; the canula was now withdrawn, leaving the tube in the position occupied by it. Two small holes in that portion of the tube corresponding with the interior of the cyst, permitted the escape of the liquid and the injection of fluids at will. As a full report of this case would require some pages, I will give in a few words the result:—

Nothing unpleasant followed the operation; ten days after, iodine was injected—no unpleasant symptoms. For the first month the tumor was gradually reduced in size; after this the discharges became purulent, and the cyst ceased to diminish. Two months after the operation, she left the hospital—the purulent discharge continuing. Ten days after her departure she returned, and in a few minutes after entering the hospital, to the great surprise of every one, she was delivered of a child at five months. Peritonitis and death, in a few days, was the result. The post mortem revealed a rupture of the sack, but not at one of the openings, as would have been supposed, but at the superior portion of the cyst. The premature delivery was the result of this rupture. The operation had nothing to do with the unpleasant result. I should have stated that the patient was presented to the Academie some twenty days after the operation—no one suspecting, for a moment, that she was pregnant.

The discussion which followed was not so much upon the mode of operating, as to whether we should attempt the radical cure by an operation, that is, by the injection of iodine and other irritants.

MM. Malgaigne, Moreau, Huguier, Cazeaux, and Velpeau, entered warmly into the discussion. The discussion is to be continued, and in my next letter I will at least give you the position of these distinguished men upon this much vexed question. At present, suffice it to say, that the remarks of MM. Velpeau and Malgaigne were, notwithstanding the brilliant statistics of Dr. Atlee, by no means complimentary to our American surgeons, who have proposed and practiced the extirpation of ovarian tumors.

As ever yours, &c.

W. F. WESTMORELAND.

—*Atlanta Medical and Surgical Journal.*

